# Subject-specific features of excitation/inhibition profiles in neurodegenerative diseases

**DOI:** 10.3389/fnagi.2022.868342

**Published:** 2022-08-05

**Authors:** Anita Monteverdi, Fulvia Palesi, Alfredo Costa, Paolo Vitali, Anna Pichiecchio, Matteo Cotta Ramusino, Sara Bernini, Viktor Jirsa, Claudia A. M. Gandini Wheeler-Kingshott, Egidio D’Angelo

**Affiliations:** ^1^Brain Connectivity Center, IRCCS Mondino Foundation, Pavia, Italy; ^2^Department of Brain and Behavioral Sciences, University of Pavia, Pavia, Italy; ^3^Unit of Behavioral Neurology, IRCCS Mondino Foundation, Pavia, Italy; ^4^Department of Radiology, IRCCS Policlinico San Donato, Milan, Italy; ^5^Department of Biomedical Sciences for Health, University of Milan, Milan, Italy; ^6^Advanced Imaging and Radiomic Center, IRCCS Mondino Foundation, Pavia, Italy; ^7^Dementia Research Center, IRCCS Mondino Foundation, Pavia, Italy; ^8^Institut de Neurosciences des Systèmes, INSERM, INS, Aix-Marseille University, Marseille, France; ^9^NMR Research Unit, Department of Neuroinflammation, Queen Square MS Centre, University College London (UCL) Queen Square Institute of Neurology, London, United Kingdom

**Keywords:** brain dynamics, excitatory/inhibitory balance, Alzheimer’s Disease, Frontotemporal Dementia, Amyotrophic Lateral Sclerosis, MRI, connectivity

## Abstract

Brain pathologies are characterized by microscopic changes in neurons and synapses that reverberate into large scale networks altering brain dynamics and functional states. An important yet unresolved issue concerns the impact of patients’ excitation/inhibition profiles on neurodegenerative diseases including Alzheimer’s Disease, Frontotemporal Dementia, and Amyotrophic Lateral Sclerosis. In this work, we used The Virtual Brain (TVB) simulation platform to simulate brain dynamics in healthy and neurodegenerative conditions and to extract information about the excitatory/inhibitory balance in single subjects. The brain structural and functional connectomes were extracted from 3T-MRI (Magnetic Resonance Imaging) scans and TVB nodes were represented by a Wong-Wang neural mass model endowing an explicit representation of the excitatory/inhibitory balance. Simulations were performed including both cerebral and cerebellar nodes and their structural connections to explore cerebellar impact on brain dynamics generation. The potential for clinical translation of TVB derived biophysical parameters was assessed by exploring their association with patients’ cognitive performance and testing their discriminative power between clinical conditions. Our results showed that TVB biophysical parameters differed between clinical phenotypes, predicting higher global coupling and inhibition in Alzheimer’s Disease and stronger N-methyl-D-aspartate (NMDA) receptor-dependent excitation in Amyotrophic Lateral Sclerosis. These physio-pathological parameters allowed us to perform an advanced analysis of patients’ conditions. In backward regressions, TVB-derived parameters significantly contributed to explain the variation of neuropsychological scores and, in discriminant analysis, the combination of TVB parameters and neuropsychological scores significantly improved the discriminative power between clinical conditions. Moreover, cluster analysis provided a unique description of the excitatory/inhibitory balance in individual patients. Importantly, the integration of cerebro-cerebellar loops in simulations improved TVB predictive power, i.e., the correlation between experimental and simulated functional connectivity in all pathological conditions supporting the cerebellar role in brain function disrupted by neurodegeneration. Overall, TVB simulations reveal differences in the excitatory/inhibitory balance of individual patients that, combined with cognitive assessment, can promote the personalized diagnosis and therapy of neurodegenerative diseases.

## Introduction

Neuroscience is showing a growing interest in merging results at different scales of complexity to achieve a global and comprehensive knowledge of the brain and its mechanisms. In this context, brain modeling can be used to bridge the gap between cellular phenomena and whole-brain dynamics, both in physiological (i.e., healthy) and pathological conditions (D’Angelo and Gandini Wheeler-Kingshott, 2017). The Virtual Brain (TVB) (Sanz-Leon et al., 2013, 2015) is a neuroinformatic platform recently developed to simulate brain dynamics starting from individual structural and functional connectivity (SC and FC, respectively) matrices constructed from MRI (magnetic resonance imaging) data. TVB has been used to characterize brain dynamics in healthy subjects (Schirner et al., 2018) but also to explore pathological mechanisms in neurological diseases, such as epilepsy (Jirsa et al., 2017), stroke (Falcon et al., 2016), brain tumor (Aerts et al., 2018), and Alzheimer’s Disease (Zimmermann et al., 2018; Stefanovski et al., 2019).

Neurodegenerative pathologies ranging from Alzheimer’s Disease, Frontotemporal Dementia, and Amyotrophic Lateral Sclerosis are reportedly characterized by a disrupted balance between excitation and inhibition.

Glutamate and GABA concentrations are relevant to the excitatory/inhibitory balance. An increase or decrease in their concentrations can lead to hyper/hypo excitation or inhibition, possibly contributing to neurodegeneration. Indeed, hyperexcitation is thought to play a pivotal role in Alzheimer Disease, Frontotemporal Dementia, and Amyotrophic Lateral Sclerosis pathogenesis (Benussi et al., 2019; Maestú et al., 2021; Pradhan and Bellingham, 2021), but multiform and sometimes contradictory results based on empirical observations make it difficult to gain an overall agreement on the neural mechanisms and the evolution of hyperexcitation over the course of the disease. In addition, despite some controversies, increasing findings are supporting GABAergic remodeling as an important feature of Alzheimer’s Disease condition (Bi et al., 2020). GABAergic dysfunction is less explored in Frontotemporal Dementia and Amyotrophic Lateral Sclerosis, but it has been demonstrated that baseline GABA levels can influence response to therapies in Frontotemporal Dementia patients (Adams et al., 2021) while impaired cortical inhibition due to GABAergic dysfunction can affect Amyotrophic Lateral Sclerosis progression (Zanette et al., 2002). Predicting treatment effectiveness for Alzheimer’s Disease, Frontotemporal Dementia, and Amyotrophic Lateral Sclerosis patients remains problematic, and the lack of meaningful biomarkers for patients’ classification worsen the situation.

Despite the importance of excitatory/inhibitory balance disruption in pathologies, excitation/inhibition experimental determination, e.g., evaluating GABA and glutamate concentrations, in single subjects is yet to reach clinical adoption because of longer acquisition times, the need for spectral editing techniques not implemented on clinical scanners and the lack of community guidelines and protocols standardization. Since TVB is designed to extract information about connectivity and network parameters including those linked to inhibition/excitation pathways in single human subjects, starting from data that can be acquired with clinical scanners, it has a true potential to foster personalized and precision medicine, especially in those neurodegenerative conditions mentioned above.

It is important also to highlight that TVB analysis should include not only cerebral nodes and their structural connections to one another, but also the cerebellum. Recently, it was shown that integrating cerebro-cerebellar connections can improve TVB predictive capability in healthy subjects (Palesi et al., 2020). This is in line with the increasing evidence supporting cerebellar involvement not only in motor learning and coordination (D’Angelo, 2019) but also in cognitive processing (Timmann and Daum, 2007; Timmann et al., 2010; Castellazzi et al., 2018; Casiraghi et al., 2019). Cerebellar impairment has been revealed in Alzheimer’s Disease (Castellazzi et al., 2014; Jacobs et al., 2018; Palesi et al., 2018), Frontotemporal Dementia (Pizzarotti et al., 2020), and Amyotrophic Lateral Sclerosis (Prell and Grosskreutz, 2013).

In this work, we exploited TVB capabilities to (i) characterize each group of subjects by providing personalized excitation/inhibition profiles and (ii) assess the cerebellar impact on brain dynamics generation in Healthy Controls and in Alzheimer’s Disease, Frontotemporal Dementia, and Amyotrophic Lateral Sclerosis. TVB simulations were performed using the Wong-Wang model (Deco et al., 2014), which allowed us to derive a set of subject-specific biophysical parameters able to describe global brain dynamics and the excitatory/inhibitory balance in local networks. We evaluated the potential for clinical translation of the biophysical parameters obtained from TVB simulations by exploring their association with patients’ cognitive performance and testing their discriminative power between clinical conditions and neuropsychological domains. This work, overall, can contribute to the progress of personalized and precision medicine by providing a unique description of the excitatory/inhibitory balance at single-subject level, opening new perspectives for brain modeling in neurodegenerative diseases.

## Materials and methods

**FIGURE 1:**
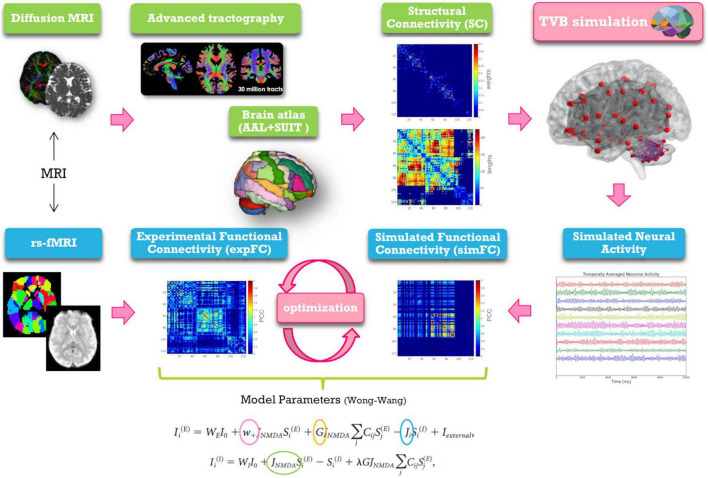
Schematic representation of modeling workflow. MRI is used to obtain *the structural and experimental functional connectivity matrices* (SC and expFC) needed for TVB construction and optimization. From top left, clockwise: diffusion weighted images are preprocessed and elaborated to yield whole-brain tractography. An *ad hoc* parcellation atlas combining AAL atlas and SUIT is used to map the SC matrices obtained from whole-brain tractography parcellation (top weight matrix, bottom distance matrix). The Virtual Brain (TVB) is constructed using the SC matrix for edges and neural masses for nodes. TVB simulations of neural activity allow to extract BOLD signals for each node leading to define the *simulated functional connectivity* (simFC) *matrix*. TVB optimization is performed through model inversion by comparing the simFC with the expFC. Model parameters, highlighted with circles, and the corresponding equations are shown at the bottom.

**FIGURE 2:**
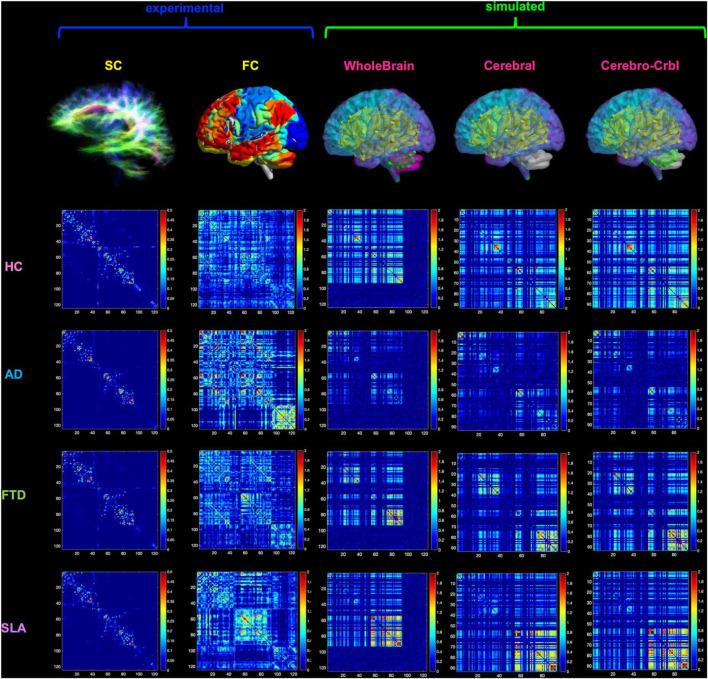
Networks connectivity matrices. Columns 1 and 2 show the experimental structural (SC) and functional connectivity (FC) matrices, which were used as input for TVB simulations in four different groups: healthy (HC), Alzheimer’s Disease (AD), Frontotemporal Dementia (FTD), and Amyotrophic Lateral Sclerosis (ALS). For each group, matrices of a randomly chosen subject are reported as an example. Columns 3–5 show the simulated FC obtained at single-subject level with three different networks: whole-brain, cortical subnetwork (Cerebral) and embedded cerebro-cerebellar subnetwork (Cerebro-Crbl). In the whole-brain network simulations were performed using whole-brain nodes and connections (whole-brain nodes and edges are colored); in the cortical subnetwork only cerebral cortex nodes and connections were considered (cortical nodes and edges are colored); in the embedded cerebro-cerebellar subnetwork cerebral cortex nodes were considered taking into account the influence of cerebro-cerebellar connections (cortical nodes and cerebellar edges are colored).

In this work individual’s subject analysis was conducted as described in Figure 1 and simulations were performed in three networks (Palesi et al., 2020): whole-brain network, cortical subnetwork, and embedded cerebro-cerebellar subnetwork (see section networks, Figure 2).

### Subjects

Sixty patients affected by neurodegenerative diseases were recruited at the IRCCS Mondino Foundation, as part of a study on cognitive impairment published in Palesi et al. (2018); Castellazzi et al. (2020), Lorenzi et al. (2020), and Pizzarotti et al. (2020). The study was carried out in accordance with the Declaration of Helsinki with written informed consent from all subjects. The protocol was approved by the local ethic committee of the IRCCS Mondino Foundation. Patients underwent a complete diagnostic workup including neuropsychological assessment, MRI (and electroneuromyography in patients with motor neuron impairment) to obtain an exhaustive phenotypic profiling and a correct etiological definition of each subject. Based on the most recent diagnostic criteria subjects were classified into three groups: 15 Alzheimer’s Disease patients (McKhann, 2012) (6 females, 70 ± 7 years), 15 Frontotemporal Dementia patients (4 females, 69 ± 7 years) [including behavioral Frontotemporal Dementia (Rascovsky and Grossman, 2014) and Primary Progressive Aphasia (Gorno-Tempini et al., 2011)], 15 Amyotrophic Lateral Sclerosis (de Carvalho et al., 2011) patients (7 females, 67 ± 8 years). In detail, diagnosis of Alzheimer’s Disease was made according to the criteria of the National Institute of Neurological and Communicative Disorders and Stroke and Alzheimer’s Disease and Related Disorders Association (NINCDS-ADRDA) workgroup (McKhann, 2012); Frontotemporal Dementia diagnosis was supported according to Rascovsky diagnostic criteria but not determined by the cognitive profile and no patient was excluded based on neuropsychological profile if diagnostic criteria were still met; Amyotrophic Lateral Sclerosis diagnosis was made in patients fulfilling Awaji criteria (de Carvalho et al., 2011) and this group included patients with Amyotrophic Lateral Sclerosis and mild cognitive impairment. In addition, 15 Healthy Controls (8 females, 64 ± 11 years) were enrolled on a voluntary basis as reference group. All Healthy Controls underwent clinical assessment to exclude any cognitive or motoneuron impairment. For all subjects, exclusion criteria were: age > 80 years, a diagnosis of significant medical, neurological (other than Alzheimer’s Disease, Frontotemporal Dementia, Amyotrophic Lateral Sclerosis) and psychiatric disorder, pharmacologically treated delirium or hallucinations and secondary causes of cognitive decline (e.g., vascular metabolic, endocrine, toxic, and iatrogenic). Table 1 shows demographic, clinical, and neuropsychological data.

**TABLE 1:** Demographics, clinical, and neuropsychological data (means, SDs, and group differences).

Measures	HC	AD	FTD	ALS	*P*-value
Males/females	7/8	9/6	11/4	8/7	0.201
Age (years)	64 (11)	70 (7)	69 (7)	67 (8)	0.494
Education (years)	10 (3)	8 (3)	10 (4)	9 (5)	−
Memory	3.0 (0.4)	0.7 (0.7)	1.6 (0.7)	2.6 (0.6)	<0.001*
Executive-function	2.9 (0.6)	0.8 (1.1)	1.0 (0.9)	1.9 (0.9)	<0.001*
Attention	3.4 (0.5)	1.1 (1.0)	1.3 (1.2)	2.0 (0.8)	<0.001*
Language	3.5 (0.5)	1.1 (1.3)	1.3 (1.0)	2.9 (1.2)	<0.001*
Visuospatial skills	3.7 (0.8)	1.2 (1.7)	2.2 (1.9)	2.2 (2.0)	0.002*

Gender, age, education, and neuropsychological scores are reported for each group (HC, Healthy Controls; AD, Alzheimer’s Disease; FTD, Frontotemporal Dementia; ALS, Amyotrophic Lateral Sclerosis) as mean values and standard deviations in brackets. Significant threshold is set at p < 0.05.

*Refers to significant group differences assessed with Kruskal-Wallis.

### Neuropsychological examination

All subjects underwent a neuropsychological examination based on a standardized battery of tests to assess their global cognitive status [Mini-Mental State Examination (MMSE); Folstein et al., 1975] and different cognitive domains: attention [Stroop test (Caffarra et al., 2002a), Trail Making test A and B (Giovagnoli et al., 1996), Attentive Matrices (Spinnler, 1987)], memory [Digit and Verbal span, Corsi block-tapping test, Logical Memory test (Spinnler, 1987), Rey-Osterrieth complex figure delayed recall (Caffarra et al., 2002b), Rey’s 15 words test (Carlesimo et al., 1996)], language [phonological (Carlesimo et al., 1996) and semantic (Novelli et al., 1986) verbal fluency], logical-executive functions [(Raven’s Matrices 1947; Carlesimo et al., 1996), Winconsing Card Sorting test (Laiacona et al., 2000), Frontal Assessment Battery (Appollonio et al., 2005)] and visuospatial skills [Rey-Osterrieth complex figure copy (Caffarra et al., 2002b)]. For each test age-, gender-, and education-corrected scores were computed and then transformed into equivalent scores ranging from 0 (pathological) to 4 (normal) on the basis of the equivalent score standardization method (Capitani and Laiacona, 1997). For each cognitive domain, a weighted score was derived from the average of the equivalent scores of the tests belonging to that specific cognitive domain.

### Magnetic resonance imaging acquisitions

All subjects underwent MRI examination using a 3T Siemens Skyra scanner with a 32-channel head coil. The protocol included resting-state fMRI (T_2_*-weighted GRE-EPI sequence, TR/TE = 3,010/20 ms; 60 slices, acquisition matrix = 90 × 90, voxel size = 2.5 × 2.5 × 2.5 mm^3^ isotropic, 120 volumes) and diffusion weighted (DW) imaging [SE-EPI sequence, TR/TE = 10,000/97 ms, 70 slices with no gap, acquisition matrix = 120 × 120, voxel size = 2 × 2 × 2 mm^3^ isotropic, 64 diffusion-weighted directions, *b*-value = 1,200 s/mm^2^, 10 volumes with no diffusion weighting (b_0_ image)]. For anatomical reference, a whole brain high-resolution 3D sagittal T1-weighted scan [3DT1 sequence, TR/TE = 2,300/2.95 ms, TI = 900 ms, flip angle = 9°, 176 slices, acquisition matrix = 256 × 256, in-plane resolution = 1.05 × 1.05 mm^2^, slice thickness = 1.2 mm] was also acquired.

### Preprocessing and tractography of diffusion data

For each subject, a mean b_0_ image was obtained averaging the 10 volumes acquired with no diffusion weighting. DW data were denoised and corrected for Gibbs artifact (Tournier et al., 2019) and eddy current distortions, and aligned to the mean b_0_ image using the eddy tool [FMRIB Software Library (FSL)^1^; Andersson and Sotiropoulos, 2016]. A binary brain mask was obtained from the mean b_0_ image using the brain extraction tool (Smith et al., 2006) and DTIFIT algorithm (FSL) was used to generate individual fractional anisotropy (FA) and mean diffusivity (MD) maps. 3DT1-weighted images were segmented using MRtrix3^2^ (Patenaude et al., 2011) as white matter (WM), gray matter (GM), subcortical GM, and cerebrospinal fluid (CSF) masks. 30 million streamlines whole-brain anatomically constrained tractography (Smith et al., 2012) was performed with MRtrix3, estimating fibers orientation distribution with multi-shell multi-tissue constrained spherical deconvolution (CSD) and using probabilistic streamline tractography (Tournier et al., 2012). As in previous works (Palesi et al., 2017, 2020), a correction of spurious cerebro-cerebellar tracts was performed excluding the ipsilateral connections from the whole-brain tractogram.

### Preprocessing of fMRI data

fMRI preprocessing was carried out combining SPM12^3^, FSL and MRtrix3 commands in a custom MATLABR2019b script. Marchenko-Pastur principal component analysis (MP-PCA) denoising (Ades-Aron et al., 2020) was firstly performed, followed by slice-timing correction, realignment to the mean functional volume and affine registration to the 3DT1-weighted volume. These steps were followed by a polynomial detrend and a 24 motion parameters regression (Friston et al., 1996). A subject-specific CSF mask was extracted from the 3DT1 segmentation, eroded using a 99% probability threshold, and constrained to areas within the ALVIN (Automatic Lateral Ventricle delIneatioN) mask of the ventricles (Kempton et al., 2011). These corrections were performed to avoid the risk of capturing signals of interest from adjacent GM voxels, and nuisance regressors identified within the restricted CSF mask were removed using a component-based noise correction (compCor) approach (Behzadi et al., 2007; Muschelli et al., 2014). Temporal band-pass filtering (0.008–0.09 Hz) was finally applied.

### Structural and functional connectivity

Connectomes of SC and FC were estimated combining a parcellation atlas with whole-brain tractography and rs-fMRI signals of each subject, respectively. An *ad hoc* GM parcellation atlas was created combining 93 cerebral (including cortical and deep GM structures) and 31 cerebellar (SUIT, A spatially unbiased atlas template of the cerebellum and brainstem) labels (Diedrichsen et al., 2009) in MNI152 space. Each GM parcellation was considered as a node for the connectivity analysis. The atlas was transformed to subject-space inverting the normalization from the 3DT1-weighted volume to the MNI152 standard space. The parcellation atlas applied to the whole-brain tractography led to two types of SC matrices: a distance matrix containing the length of tracts connecting each pair of nodes, and a weight matrix in which connections strengths (number of streamlines) were normalized by the maximum value per each subject. The time-course of BOLD signals was extracted for each node and the experimental FC matrix (expFC) was computed as the Pearson’s correlation coefficient (PCC) of the time-course between each pair of brain regions. Matrix elements were converted with a Fisher’s z transformation and thresholded at 0.1206 (Palesi et al., 2020).

### Brain dynamics simulation with the virtual brain

TVB workflow includes several steps: (1) incorporation of subject SC matrices; (2) selection of a mean field/neural mass mathematical model; (3) simulation of the rs-fMRI time-course per node and creation of the simulated FC matrix (simFC); (4) model parameters tuning to achieve the best matching between simFC and expFC matrices; (5) final simulation of brain dynamics with the optimal model parameters as described in detail by Deco et al. (2014).

#### Computational model from neuronal activity to large-scale signals

The Wong-Wang model (Deco et al., 2014) implemented as highly optimized C code (Schirner et al., 2021) was chosen to simulate whole-brain dynamics. This dynamic mean field model simulates the local regional neuronal activity as the result of a network composed of interconnected excitatory and inhibitory neurons coupled through NMDA and GABA receptor types. Details of the Wong-Wang model can be found in Deco et al. (2014). Briefly, brain dynamics are described by the following set of coupled non-linear stochastic differential equations:


(1) $$I_{i}^{\left(E\right)}=W_{E}\,I_{0}+w_{+}\,J_{N\,M\,D\,A}\,S_{i}^{\left(E\right)}+G\,J_{N\,M\,D\,A}\,\sum_{j}C_{i\,j}\,S_{j}^{\left(E\right)}-J_{i}\,S_{i}^{\left(I\right)}$$

(2) $$I_{i}^{\left(I\right)}=W_{I}\,I_{0}+J_{N\,M\,D\,A}\,S_{i}^{\left(E\right)}-S_{i}^{\left(I\right)}$$

(3) $$r_{i}^{\left(E\right)}=H^{\left(E\right)}\,\left(I_{i}^{\left(E\right)}\right)=\frac{a_{E}\,I_{i}^{\left(E\right)}-b_{E}}{1-\exp\left(-d_{E}\,\left(a_{E}\,I_{i}^{\left(E\right)}-b_{E}\right)\right)}$$

(4) $$r_{i}^{\left(I\right)}=H^{\left(I\right)}\,\left(I_{i}^{\left(I\right)}\right)=\frac{a_{I}\,I_{i}^{\left(I\right)}-b_{I}}{1-\exp\left(-d_{I}\,\left(a_{I}\,I_{i}^{\left(I\right)}-b_{I}\right)\right)}$$

(5) $$\frac{d\,S_{i}^{\left(E\right)}\,\left(t\right)}{d\,t}=-\frac{S_{i}^{\left(E\right)}}{\tau_{E}}+\left(1-S_{i}^{\left(E\right)}\right)\,\gamma \,r_{i}^{\left(E\right)}+\sigma \,v_{i}\,\left(t\right)$$

(6) $$\frac{d\,S_{i}^{\left(I\right)}\,\left(t\right)}{d\,t}=-\frac{S_{i}^{\left(I\right)}}{\tau_{I}}+r_{i}^{\left(I\right)}+\sigma \,v_{i}\,\left(t\right)$$

where r_i_^(E,I)^ denotes the firing rate of the excitatory (E) and inhibitory (I) populations, S_i_^(E,I)^ identifies the average excitatory or inhibitory synaptic gating variables at local area, i, and I_i_^(E,I)^ is the input current to the excitatory and inhibitory populations at local area, i. All parameters described in Supplementary Table 1 were set as in Deco et al. (2014), except those that are tuned during parameters optimization, as follows: parameter space exploration was performed for global coupling (G), which is a scaling factor denoting long-range coupling strength, and local parameters defining the strength of inhibitory (GABA) synapses (J_i_), the strength of excitatory (NMDA) synapses (J_NMDA_) and the strength of local excitatory recurrence (w_+_). Thus, this model retains information on both global brain dynamics and local excitatory/inhibitory balance and is particularly interesting for the investigation of pathological conditions.

For each set of parameters combination, resting-state BOLD fMRI time-courses were simulated over 6 min length using a Balloon-Windkessel hemodynamic neurovascular coupling model (Friston et al., 2000) and the simFC was computed as described for the expFC (see section “Structural and functional connectivity”). Parameters were adjusted iteratively until the best fit, i.e., the highest correlation, between expFC and simFC was achieved (Supplementary Figure 1).

#### Networks

To investigate the impact of the cerebellum on brain dynamics generation, simulations were performed using three different combinations of connections and nodes (Figure 2):

• Whole-brain network: whole-brain nodes and connections.
• Cortical subnetwork: cerebral cortex nodes and connections (excluding cerebro-cerebellar connections).
• Embedded cerebro-cerebellar subnetwork: cerebral cortex nodes but also considering cerebellar nodes and hence the cerebro-cerebellar connections.

For each of these three networks predictive power was evaluated as the mean PCC between expFC and simFC matrices in different clinical conditions (Healthy Controls, Alzheimer’s Disease, Frontotemporal Dementia, Amyotrophic Lateral Sclerosis).

### Statistic

Statistical tests were performed using SPSS software version 21 (IBM, Armonk, New York, United States).

#### Excitation/inhibition role in neurodegeneration

To assess whether biophysical parameters derived from TVB differ according to the clinical condition, optimal model parameters were tested for normality (Shapiro-Wilk test) and differences between groups were assessed with non-parametric tests (Kruskal-Wallis across all groups and Mann-Whitney between each pair of groups) when they did not present a Gaussian distribution.

A multiple regression analysis was performed to investigate the relationship between individual scores of the 5 cognitive domains (attention, memory, language, logical-executive functions, visuospatial skills) and the optimal model parameters. Neuropsychological scores in each cognitive domain were considered as dependent variables while model parameters combined with age, gender, and group category were used as predictors in a backward approach. The regression algorithm automatically removed one or more predictors to identify which of them significantly (*p* < 0.05) explained neuropsychological scores variance in all subjects together.

Moreover, to assess the relevance of these parameters in discriminating between normal and pathological conditions, a discriminant analysis was performed using the group as the dependent variable and considering as independent variables, in turns: (i) model parameters alone, (ii) neuropsychological scores alone, and (iii) a combination of both. To visualize and assess the sensitivity and specificity of the best discriminative variables, receiving operating characteristics (ROC) curves and corresponding areas under the curve (AUC) were calculated.

Finally, a k-mean cluster analysis was performed to reconstruct subjects-specific excitation/inhibition profiles. The number of clusters was an input parameter, arbitrarily set equal to 4, matching the number of variables considered (i.e., the 4 model parameters). A frequency analysis of the clusters in terms of clinical groups enabled us to reach a deeper understanding of their respective excitatory/inhibitory balance profiles. This percentage of subjects belonging to one of the clusters in each clinical group was used to qualitatively describe the occurrence of specific patterns of excitatory/inhibitory balance in the population.

#### Cerebellar role in brain dynamics in neurodegeneration

PCC obtained with the three networks were normally distributed (Shapiro-Wilk test), thus parametric tests were used to compare them between different conditions. First, to assess TVB predictive power for each clinical condition, a one-way ANOVA was performed between the PCC of each network across groups (Healthy Controls, Alzheimer’s Disease, Frontotemporal Dementia, Amyotrophic Lateral Sclerosis). Then, to assess the impact of each specific network on TVB predictive power, a multivariate general linear model (GLM) with Bonferroni correction was chosen to compare PCC values of the three networks within each group.

### Code and data accessibility

All codes used for this study are freely available. The optimized TVB C code can be found at https://github.com/BrainModes/fast_tvb. Dataset will be available at 10.5281/zenodo.5796063.

## Results

### Excitation/inhibition role in neurodegeneration

Both global (G) and local (J_i_, J_NMDA_, w_+)_ parameters were adjusted iteratively to optimize the model fit to empirical data. Optimal model parameters were found across the whole-brain network of each subject.

#### Group differences in the virtual brain parameters

**FIGURE 3:**
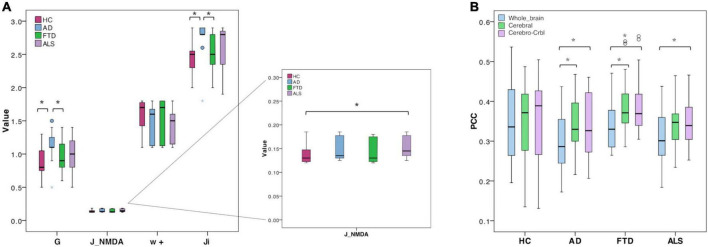
Boxplots of optimal biophysical parameters and Pearson correlation coefficients (PCC). **(A)** Boxplots of optimal biophysical parameters derived from TVB (global coupling, G, excitatory synaptic coupling, J_NMDA, local excitatory recurrence, w+, inhibitory synaptic coupling, Ji) across groups (Healthy Controls, Alzheimer’s Disease, Frontotemporal Dementia, and Amyotrophic Lateral Sclerosis). The asterisk (*) indicates a significant difference (Mann-Whitney, *p* < 0.05) between groups (see Table 2 for details). **(B)** Boxplots of Pearson correlation coefficients (PCC) between experimental and simulated FC for all groups (Healthy Controls, Alzheimer’s Disease, Frontotemporal Dementia, Amyotrophic Lateral Sclerosis) and networks (whole-brain network, Whole_brain, cortical subnetwork, cerebral; embedded cerebro-cerebellar subnetwork, Cerebro-Crbl). Asterisks (*) indicate a significant difference (*p* < 0.05) between networks (see Table 2 for details).

The biophysical parameters derived from TVB were compared between groups to assess whether, at group level, their value could differ according to the clinical condition. Both global and local biophysical parameters showed significant differences between groups (Table 2 and Figure 3A): Alzheimer’s Disease patients showed higher G and J_i_ compared to Healthy Controls and Frontotemporal Dementia (*p* < 0.05); Amyotrophic Lateral Sclerosis patients showed higher J_NMDA_ than Healthy Controls (*p* < 0.05); no statistically significant differences were found when comparing Healthy Controls and Frontotemporal Dementia with other groups.

**TABLE 2:** Optimal model parameters and Pearson correlation coefficients per group.

TVB_parameters	HC	AD	FTD	ALS	*P*-value^a^
	Mean (*SD*)	Mean (*SD*)	Mean (*SD*)	Mean (*SD*)	
G	0.887 (0.226)	0.137 (0.236)	0.980 (0.248)	0.993 (0.281)	0.047*°
J_i_	2.473 (0.268)	2.753 (0.275)	2.520 (0.293)	2.580 (0.371)	0.047*°
J_NMDA_	0.137 (0.020)	0.147 (0.024)	0.143 (0.026)	0.152 (0.023)	0.106^◊^
w_+_	1.587 (0.238)	1.477 (0.280)	1.527 (0.312)	1.430 (0.247)	0.274

**PCC**	**Mean (*SD*)**	**Mean (*SD*)**	**Mean (*SD*)**	**Mean (*SD*)**	

Whole-brain	0.342 (0.010)	0.297 (0.076)	0.343 (0.064)	0.312 (0.070)	0.285
Cerebral	0.347 (0.097)	0.342 (0.077)	0.392 (0.080)	0.341 (0.056)	0.241
Cerebro-Crbl	0.353 (0.109)	0.337 (0.087)	0.396 (0.084)	0.348 (0.061)	0.283
*p*-value^b^	0.725	<0.001^⋅^^†^	<0.001^⋅^^†^	0.022^⋅^^†^	

Model optimal biophysical parameters (G, global coupling; J_NMDA_, excitatory synaptic coupling; w_+_, local excitatory recurrence; J_i_, inhibitory synaptic coupling) and Pearson Correlation Coefficients (PCC) between experimental and simulated FC for all groups (HC, Healthy Controls; AD, Alzheimer’s Disease; FTD, Frontotemporal Dementia; ALS, Amyotrophic Lateral Sclerosis) and networks (whole-brain, cerebral subnetwork, and embedded cerebro-cerebellar subnetwork = Cerebro-Crbl). Values are expressed as mean values and standard deviation in brackets. Significant threshold is set at p < 0.05.

^a^Group differences assessed with Kruskal-Wallis for optimal model parameters and one-way ANOVA for PCC.

^b^PCC differences between networks assessed with GLM.

*Refers to significant difference between Healthy Controls and Alzheimer’s Disease assessed with Mann-Whitney.

°Refers to significant difference between Alzheimer’s Disease and Frontotemporal Dementia assessed with Mann-Whitney.

^◊^Refers to significant difference between Healthy Controls and Amyotrophic Lateral Sclerosis assessed with Mann-Whitney.

^⋅^Refers to p < 0.003 between whole-brain and cortical networks.

^†^Refers to p < 0.01 between whole-brain and embedded networks.

#### Relationship between the virtual brain parameters and neuropsychological scores

Parameters used in backward regressions, significantly explained the variation of scores in different neuropsychological domains. The explained variance of each neuropsychological domain was progressively reduced by simplifying the regression model through the removal of one or more predictors and ranged from ∼20 to ∼8%. For each cognitive domain, a different combination of features was necessary to significantly (*p* < 0.05) explain a percentage of the variance (Table 3).

**TABLE 3:** Backward regressions results.

	Predictors	Explained variance	Significance
Memory	Ji	8.4%	0.028
Executive-function	Group, gender, age, w+, G Group, gender, age, G	19.9% 19.8%	0.037 0.018
	Group, age, G	19.5%	0.008
	Group, age	18.5%	0.004
Attention	Group, Ji, gender, age	16.9%	0.040
	Group, Ji, age	16.7%	0.019
	Group, Ji	15%	0.011
	Group	12%	0.008
Language	Group, G	10.8%	0.044
	G	8.7%	0.025
Visuospatial skills	Group, Ji	10.7%	0.045

The variance explained by the parameters used in backward regressions is calculated with the R^2^ index. Significant threshold is set at p < 0.05. For each cognitive domain a different combination of features significantly explains a percentage of the variance (ANOVA).

#### Discriminative power of the virtual brain parameters and neuropsychological scores

**FIGURE 4:**
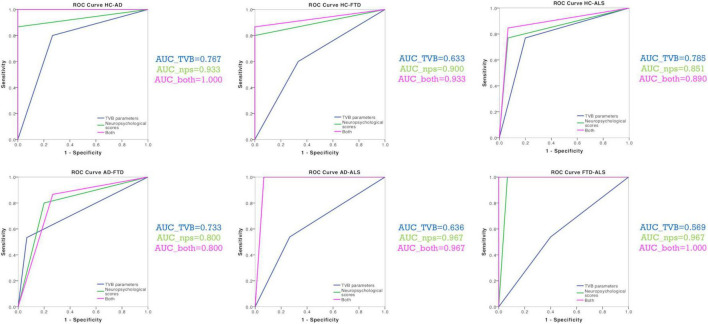
Classification analysis. ROC curves were calculated for each classification (Healthy Controls vs. Alzheimer’s Disease, Healthy Controls vs. Frontotemporal Dementia, Healthy Controls vs. Amyotrophic Lateral Sclerosis, Alzheimer’s Disease vs. Frontotemporal Dementia, Alzheimer’s Disease vs. Amyotrophic Lateral Sclerosis, Frontotemporal Dementia vs. Amyotrophic Lateral Sclerosis) with their corresponding groups of variables (TVB parameters alone, neuropsychological scores alone, TVB parameters combined with neuropsychological scores). AUC values confirmed that TVB parameters alone (blue) always yielded a poorer discriminant power than that offered by neuropsychological scores alone (green). The combination of TVB parameters with neuropsychological scores improved the discriminative power in all classifications reaching 100% when distinguishing between Alzheimer’s Disease and Healthy Controls and between Frontotemporal Dementia and Amyotrophic Lateral Sclerosis.

The discriminative power of TVB parameters and neuropsychological scores is reported for all comparisons (Healthy Controls vs. Alzheimer’s Disease, Healthy Controls vs. Frontotemporal Dementia, Healthy Controls vs. Amyotrophic Lateral Sclerosis, Alzheimer’s Disease vs. Frontotemporal Dementia, Alzheimer’s Disease vs. Amyotrophic Lateral Sclerosis, Frontotemporal Dementia vs. Amyotrophic Lateral Sclerosis) in Table 4. TVB parameters always yielded a poorer discriminant power (about 70%) than that offered by neuropsychological scores (about 90%). When neuropsychological scores were complemented by TVB values as joint independent variables, the discriminative power increased in all classifications reaching 100% when distinguishing between Alzheimer’s Disease and Healthy Controls, and between Frontotemporal Dementia and Amyotrophic Lateral Sclerosis. To visualize all these results, ROC curves are reported in Figure 4.

**TABLE 4:** Classification results (AUC, sensitivity and specificity) for group comparisons.

	TVB_PARAMS			NPS			TVB + NPS		
	AUC	Sens.	Spec.	AUC	Sens.	Spec.	AUC	Sens.	Spec.
HC vs. AD	76.7%	0.800	0.733	93.3%	0.867	1.000	100%	1.000	1.000
HC vs. FTD	63.3%	0.600	0.667	90%	0.800	1.000	93.3%	0.867	1.000
HC vs. ALS	76.7%	0.733	0.800	85.7%	0.769	0.933	89.3%	0.846	0.933
AD vs. FTD	73.3%	0.533	0.933	80%	0.800	0.800	80%	0.867	0.733
AD vs. ALS	66.7%	0.600	0.733	96.4%	1.000	0.933	96.4%	1.000	0.933
FTD vs. ALS	56.7%	0.533	0.600	96.4%	1.000	0.933	100%	1.000	1.000

AUC, Areas under the curve; Sens., sensitivity; Spec., specificity are reported for all classifications. (HC, Healthy Controls; AD, Alzheimer’s Disease; FTD, Frontotemporal Dementia; ALS, Amyotrophic Lateral Sclerosis) performed with different independent variables: TVB-derived biophysical parameters alone (TVB_params), neuropsychological scores alone (NPS), and a combination of both (TVB + NPS).

#### A personalized description of the excitatory/inhibitory balance

**FIGURE 5:**
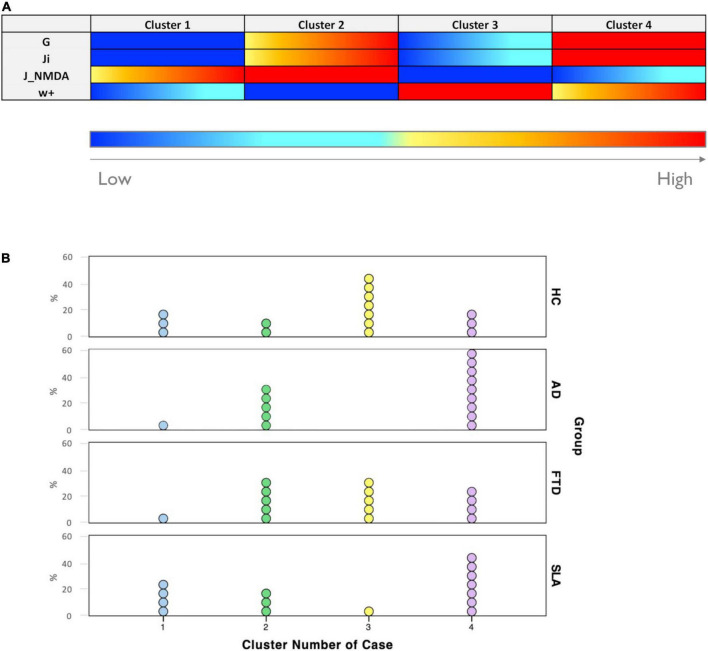
Excitation/inhibition profiles. **(A)** Each cluster was characterized by a typical excitation/inhibition profile. The color-bar (from blue to red) represents the scale from low to high of each TVB-derived biophysical parameter. **(B)** Visual representation of cluster distributions across groups (Healthy Controls, Alzheimer’s Disease, Frontotemporal Dementia, and Amyotrophic Lateral Sclerosis). Cluster numbers are reported on the *x*-axis while cluster frequencies in each condition are reported on the *y*-axis. Each dot represents a single subject.

Each of the four clusters identified with the k-means analysis was characterized by a different combination of values of TVB-derived biophysical parameters, as reported in Figure 5A and Supplementary Figure 2. Considering the biophysical meaning of each parameter derived from the simulation, we can describe the k-means clusters as follows:

• Cluster 1 is mainly characterized by medium to strong overexcitation (medium to high values of J_NMDA_).
• Cluster 2, in addition to showing strong overexcitation (very high values of J_NMDA_), is characterized by a high global coupling strength (medium to high values of G) and medium to strong overinhibition (medium to high values of J_i_).
• Cluster 3 is the only one characterized by medium or low values of G, J_i_, and J_NMDA_ and high values of local excitatory recurrence (w_+_).
• Cluster 4 is mostly characterized by overinhibition (high values of J_i_) and high global coupling strength between nodes (higher values of G).

All groups were represented in more than one clusters, as shown in Figure 5B. The distribution of subjects belonging to different condition across clusters revealed that: cluster 1 was more frequent in Healthy Controls (20%) and Amyotrophic Lateral Sclerosis (26.7%) than in Alzheimer’s Disease and Frontotemporal Dementia (both 6.7%); cluster 2 was less present in Healthy Controls (13.3%) than in pathological conditions (Alzheimer’s Disease = 33.3%, Frontotemporal Dementia = 33.3%, and Amyotrophic Lateral Sclerosis = 20%); cluster 3 was mostly composed of Healthy Controls subjects (46.7%), somewhat frequent in Frontotemporal Dementia (33.3%) and hardly present in Amyotrophic Lateral Sclerosis (6.7%) and Alzheimer’s Disease subjects (0%); cluster 4 was the most frequent in Alzheimer’s Disease (60%) and Amyotrophic Lateral Sclerosis (46.7%) and less frequent in Healthy Controls (20%) and Frontotemporal Dementia (26.7%).

### Cerebellar role in brain dynamics in neurodegeneration

To understand the role of the cerebellum in neurodegeneration, we performed TVB simulations using three different networks: (i) Whole-brain network, (ii) Cortical subnetwork, (iii) Embedded cerebro-cerebellar subnetwork as in Palesi et al. (2020). For each of these three networks, the predictive power was evaluated as the mean PCC between expFC and simFC matrices in Healthy Controls and in the pathological groups: Alzheimer’s Disease, Frontotemporal Dementia, Amyotrophic Lateral Sclerosis.

#### Predictive power of the virtual brain simulations

TVB simulation performed both in Healthy Controls and pathological conditions led to good fit values between the expFC and simFC (Table 2). No differences were found between PCC values of each network across clinical groups, but significant differences were found in each group comparing PCCs of the three networks. For all the pathological groups, PCC values obtained with the embedded cerebro-cerebellar subnetwork were significantly higher (*p* < 0.01) than those obtained with the whole-brain network, while PCCs of the cortical subnetwork were significantly higher (*p* < 0.003) than those of the whole-brain network in Alzheimer’s Disease and Frontotemporal Dementia (Table 2 and Figure 3B). No differences were found between the three networks in Healthy Controls.

## Discussion

For the first time, in this work we characterized the excitatory/inhibitory profile in neurodegenerative (Alzheimer’s Disease, Frontotemporal Dementia, Amyotrophic Lateral Sclerosis) conditions integrating cerebro-cerebellar connections in TVB. Importantly, by adopting the Wong-Wang model to model brain dynamics, we gained information on local excitatory/inhibitory balance at the single subject level.

### Excitation/inhibition role in neurodegeneration

#### Hyper-excitation and over-inhibition underly different neurodegenerative mechanisms

Parameters derived from TVB simulations using the Wong-Wang model yield information on global brain dynamics and on the local excitatory/inhibitory balance. In particular, the global scaling factor G denotes the strength of long-range connections, and higher global coupling means a greater weighting of the global over the local connectivity. The remaining three parameters define the balance between excitation and inhibition in the simulated network: J_NMDA_ represents the strength of excitatory synapses in the network, J_i_ denotes the strength of inhibitory synapses and w_+_ the strength of recurrent excitation.

Our results revealed that different clinical groups were characterized by specific TVB parameters providing new clues for the interpretation of the dysfunctional mechanisms in local microcircuits.

To date, patterns of altered FC in Alzheimer’s Disease from a resting-state networks perspective have been reported mainly in the default mode network (DMN) (Hohenfeld et al., 2018; Ibrahim et al., 2021), although a wider involvement has been suggested by our group in previous work (Castellazzi et al., 2014). Our data demonstrated that G and J_i_ were higher in Alzheimer’s Disease patients compared to Healthy Controls suggesting that Alzheimer’s Disease subjects were characterized by increased global coupling and overinhibition. This increased *G*-value in our Alzheimer’s Disease group could be interpreted as a compensatory mechanism counteracting altered cerebral connectivity, but it might also underly the hypersynchrony typically characterizing disrupted networks in patients (Castellazzi et al., 2014; Maestú et al., 2019). Furthermore, our data shows an increased inhibition suggesting that GABAergic dysfunction plays a role in Alzheimer’s Disease pathology, which is in line with the novel hypothesis that GABAergic remodeling might be an important feature of neurodegeneration (Bi et al., 2020).

It is worth noting that Alzheimer’s Disease showed higher G and J_i_ also compared to Frontotemporal Dementia patients, strengthening the tenet that the pathophysiological mechanisms underlying the two diseases are different. Indeed, Frontotemporal Dementia showed G and J_i_ values similar to Healthy Controls, consistent with the similarity of cortical neural synchronization in Frontotemporal Dementia and Healthy Controls (Nardone et al., 2018).

Finally, Amyotrophic Lateral Sclerosis patients were characterized by an increased J_NMDA_, which is in line with the cortical hyperexcitability frequently reported in this pathological condition (Pradhan and Bellingham, 2021).

#### The virtual brain-derived biophysical parameters help to explain cognitive performance

TVB-derived biophysical parameters combined with age, gender, and group category significantly explained the variance of neuropsychological scores both in Healthy Controls and pathological groups. This suggests that the levels of excitation, inhibition and global coupling are associated with cognitive performance in the different clinical conditions.

The clinical relevance of TVB parameters is further highlighted by results of the discriminant analysis. The discriminative power of neuropsychological tests alone was always higher than the one obtained with TVB parameters alone. However, when neuropsychological measures were combined with TVB parameters, the discriminative power improved, reaching in some cases 100% of accuracy. Importantly the performance of our classification was satisfactory not only to distinguish Healthy Controls from patients, but also to differentiate patients belonging to different neurodegenerative conditions. This opens an interesting perspective for the development of new diagnostic tools combining TVB parameters with neuropsychological scores in future machine learning approaches.

#### The excitatory/inhibitory balance in single-subject

For each subject, TVB predicted the optimal parameters, providing a subject-specific description of the excitatory/inhibitory balance that could be analyzed at group level (as discussed above) or used to establish cluster membership in a data-driven approach.

While at group level there were noticeable differences in excitatory/inhibitory parameters, when considering single-subjects’ profiles, K-means clusters were not group specific; this suggests an heterogeneous excitatory and inhibitory balance across subjects that could be exploited for future personalized interventions. Considering the biophysical meaning of TVB parameters, cluster 1 was mainly characterized by overexcitation and was more frequent in Healthy Controls and Amyotrophic Lateral Sclerosis. As well as being consistent with the fact that hyperexcitability is a well-known feature of Amyotrophic Lateral Sclerosis patients (Brunet et al., 2020; Pradhan and Bellingham, 2021), the presence of some Healthy Controls subjects in this cluster is not surprising. Indeed, the effect of aging on the glutamatergic system is currently under investigation (Segovia et al., 2001), and even if glutamate is mostly reported to decrease with age (Roalf et al., 2020), age-related effects on glutamatergic release and uptake processes and NMDA receptors activation could be differentially modulated in some healthy subjects. Even in cluster 2 we found some Healthy Controls, which presented not only high excitation but also high global coupling strength. This high *G*-value could be due to an increased strength of long-range connectivity or increased synchrony between nodes. It is worth noting that cluster 2 was more common in pathological conditions than in Healthy Controls, and this is in line with the frequent observation of hyperexcitation and hypersynchrony in Alzheimer’s Disease (Henstridge et al., 2019; Maestú et al., 2019, Maestú et al., 2021), Frontotemporal Dementia (Hughes et al., 2018; Benussi et al., 2019), and Amyotrophic Lateral Sclerosis (Dukic et al., 2019; Pradhan and Bellingham, 2021). Cluster 3 was the most related to Healthy Controls, while the number of patients was marginal (0 for the Alzheimer’s Disease group). This cluster is mainly characterized by high recurrent excitation; interestingly, alterations of this property are less explored as potential mechanisms in clinical conditions. Only a few network models have been developed to account for the influence of recurrent excitation and to explore its changes in pathologies. Strong self-excitation was shown to be required in network models to achieve satisfactory simulations of decision making and working memory tasks (Wong and Wang, 2006), in line with our evidence of high recurrent excitation in Healthy Controls. Moreover, in a previously proposed computational model applied to Alzheimer’s Disease the variation of local recurrent excitation has been suggested as a brain mechanism employed to compensate for alterations induced by other types of synapse loss (Bachmann et al., 2020). Unfortunately, nothing is known about recurrent excitation in network models of Frontotemporal Dementia or Amyotrophic Lateral Sclerosis, but the presence of Frontotemporal Dementia and Amyotrophic Lateral Sclerosis patients in cluster 3 prompts to explore the effect of this parameter in clinical conditions other than Alzheimer’s Disease. Finally, cluster 4 was mostly associated with Alzheimer’s Disease patients, followed by Amyotrophic Lateral Sclerosis, Frontotemporal Dementia and Healthy Controls. While convergent findings are increasingly supporting the role of a GABA function increase in Alzheimer’s Disease (Li et al., 2016; Govindpani et al., 2017; Bi et al., 2020), GABAergic dysfunction is mostly described as an overall decrease of cortical inhibition in Frontotemporal Dementia and Amyotrophic Lateral Sclerosis. Our results suggest the possibility of an increased GABAergic activity not only in Alzheimer’s Disease patients, as already observed in literature, but also in subsets of subjects affected by other neurodegenerative conditions.

It is important to point out that Frontotemporal Dementia patients appeared to be the most distributed between clusters, without a main cluster membership, and this evidence reflects the heterogeneity of our Frontotemporal Dementia cohort, which is in line with the wide spectrum of neurotransmitters deficits which has been already observed in Frontotemporal Dementia (Murley and Rowe, 2018).

### Cerebellar role in neurodegeneration

Cerebellar impairment has been consistently observed in neurodegenerative diseases, although the cerebellum has been rarely considered in neurodegenerative conditions. Disease-specific clusters of cerebellar atrophy have been found in Alzheimer’s Disease, Frontotemporal Dementia, and Amyotrophic Lateral Sclerosis (Gellersen et al., 2017; Pizzarotti et al., 2020). Functional connectivity alterations (Castellazzi et al., 2014; Jacobs et al., 2018) and WM disruption (Toniolo et al., 2020) characterize cerebro-cerebellar loops in Alzheimer’s Disease patients. Abnormal network connectivity between cerebellum and cerebral cortical regions has been confirmed in the main subtypes of Frontotemporal Dementia (Chen et al., 2019, 2020) (behavioral-variant, semantic dementia, and progressive non-fluent aphasia). In Amyotrophic Lateral Sclerosis the functional reorganization following motor neuronal loss increases cerebellar activation in motor tasks with respect to controls (Prell and Grosskreutz, 2013), while a widespread pattern of WM abnormalities has been reported together with WM volume reduction (Chen et al., 2018).

In our work, the integration of cerebro-cerebellar connections in the structural matrix improved the predictive power of TVB simulations in pathological conditions, supporting the concept of a cerebellar involvement in neurodegenerative conditions and confirming the sizeable contribution of cerebro-cerebellar connectivity to simulated brain dynamics (Palesi et al., 2020). This improvement was especially evident in Amyotrophic Lateral Sclerosis, which is a long-range motor neuron disease. This calls for future work to establish whether this result reflects a higher cerebellar recruitment determined by Amyotrophic Lateral Sclerosis functional reorganization (Prell and Grosskreutz, 2013). Studies evaluating the impact of cerebellar integration not only on static FC simulations, as performed in this work, but also on dynamic resting-state FC simulations (Hansen et al., 2015; Kong et al., 2021) are warranted.

### Study considerations

One potential concern about the present study is the small sample size. However, it should be noted that one of the most interesting aspects of TVB is to uncover subject-specific characteristics in clinical groups. In this perspective, the use of a small sample does not detract relevance from this study, also considering that other TVB applications on small cohorts can be found in literature (Aerts et al., 2018; Schirner et al., 2018). An interesting perspective would be to extend this investigation to a larger cohort of subjects and cluster TVB parameters with MRI features and other clinical data. This would allow to explore the existence of yet unrecognized categories of patients affected by dementia. Thus, while our study can be considered a first step, future studies could benefit from increasing the number of recruited subjects or analyzing open-source large-scale clinical datasets.

A technical aspect to consider is also that, in this study, whole brain parcellation was performed combining the AAL atlas with SUIT in order to account for the cerebellum. The different parcel sizes may influence structural/functional connectivity and brain dynamics (Proix et al., 2016). In this context, it is important to point out that the influence of parcellation size on topological and functional brain properties is still not fully understood. Importantly, few studies focused on this interesting issue and structural/functional organization appeared to be robust changing the anatomical parcellation atlases used, while quantitative measures (e.g., graph theoretical metrics) seemed to be modulated by the adopted parcellation approach (de Reus and van den Heuvel, 2013). Moreover, attention has never been focused on cerebro-cerebellar loops, and currently only SUIT has been validated while other cerebellar atlases are still under development. Parcellation-dependent variance in both experimental and simulated data are currently under investigation (Domhof et al., 2021).

## Conclusion

TVB-derived biophysical parameters provided a unique description of the excitatory/inhibitory balance both at group and single subject levels. An extremely high performance was achieved in patients’ discrimination combining TVB parameters and neuropsychological scores. Moreover, the integration of cerebro-cerebellar connections in TVB improved the predictive power of the model in neurodegeneration. Overall, this work opens new perspectives for the use of TVB to explore neurodegenerative mechanisms, supports the involvement of the cerebellum in determining brain dynamics in neurodegenerative diseases, and suggests a novel approach to obtain physiological information relevant to future personalized diagnosis and therapy.

## Author’s note

This manuscript has been released as a Pre-Print at BioRxiv (Monteverdi et al., 2021).

## Data availability statement

The raw data supporting the conclusions of this article will be made available by the authors, without undue reservation.

## Ethics statement

The studies involving human participants were reviewed and approved by the IRCCS Mondino Foundation. The patients/participants provided their written informed consent to participate in this study.

## Author contributions

AM performed research, analyzed data, and wrote the manuscript. FP, ED’A, and CG designed research, coordinated the work, and provided critical revision. PV and AP performed and coordinated brain MRI examinations and provided critical revision. AC, MC, and SB were involved in clinical data acquisition. VJ provided critical revision. All authors contributed to the article and approved the submitted version.

## Footnotes

1
http://fsl.fmrib.ox.ac.uk/fsl/fslwiki

2
http://www.mrtrix.org

3
https://www.fil.ion.ucl.ac.uk/spm

## Abbreviations

expFC: experimental Functional Connectivity
FC: Functional Connectivity
PCC: Pearson Correlation Coefficient
SC: Structural Connectivity
simFC: simulated Functional Connectivity
TVB: The Virtual Brain.
