# Supplementary material for: Subject-specific features of excitation/inhibition profiles in neurodegenerative diseases
Source: Front Aging Neurosci. 2022 Aug 5;14:868342. doi: 10.3389/fnagi.2022.868342 (PMC9391060; doi:10.3389/fnagi.2022.868342)
Supplement: Supplementary file 1 [file Data_Sheet_1.PDF]

Supplementary Figure 1| Parameter space exploration results. 2D parameter space heat maps show different values of correlation between experimental and simulated FC obtained at different combinations of the TVB parameters (G, Ji, J_NMDA and w+). Parameter values that yielded the highest correlation were chosen for the next steps of the analysis.

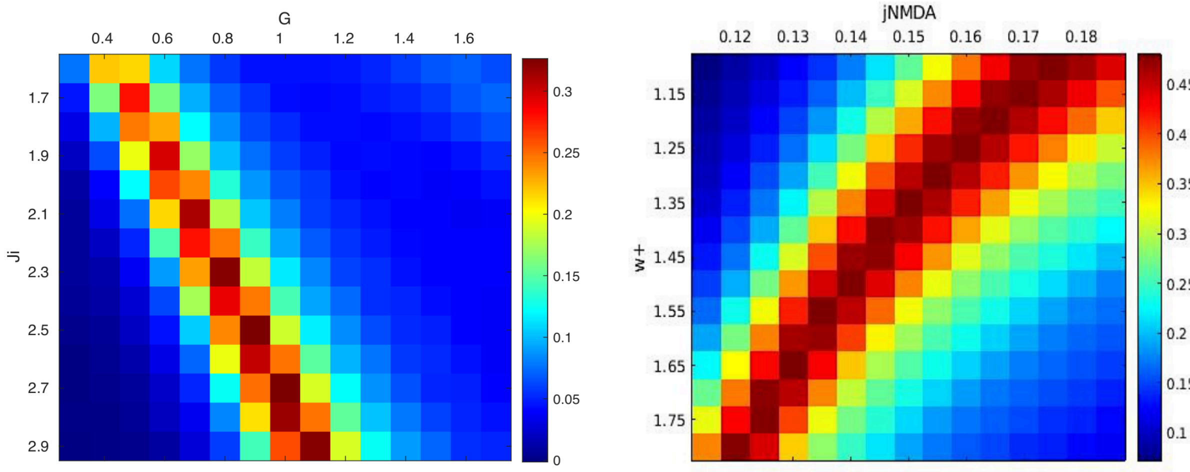

Supplementary Figure 2| Clustering analysis. (A) Visual representation of the four clusters (in different colors) identified with k-means analysis using TVB-derived optimal biophysical parameters as input variables. **(B)** Each of the four clusters was characterized by a combination of low and high values of TVB-derived biophysical parameters (Ji, J_NMDA and wp are represented on the axis and G values are represented with color code).

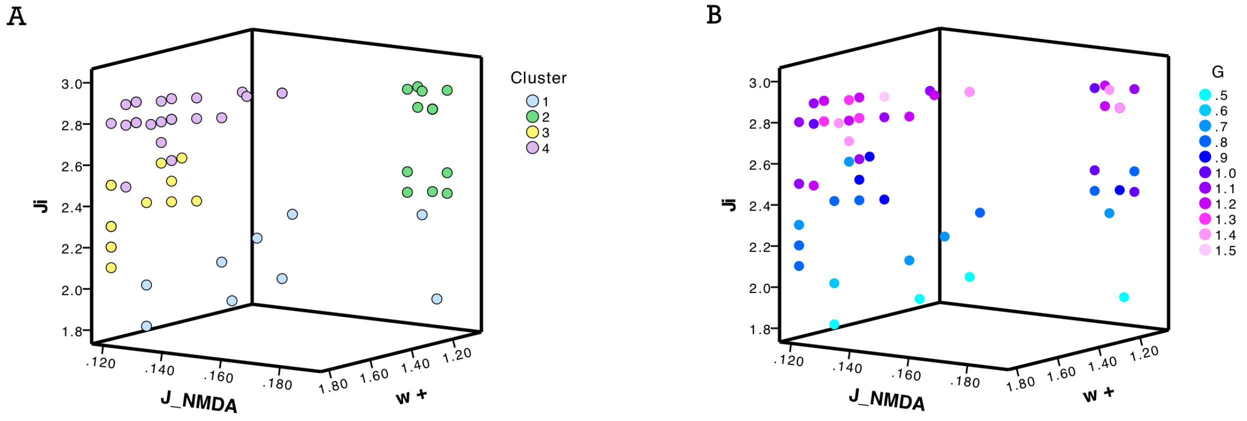

Supplementary Table 1: Wong-Wang model parameters for TVB simulations.

PARAMETERS	VALUE	DESCRIPTION
a_E, b_E, d_E, τ_E, W_E	310 nC^{-1}, 125 Hz, 0.16 s, 100 ms, 1	Excitatory gating variables
a_I, b_I, d_I, τ_I, W_I	615 nC^{-1}, 177 Hz, 0.087 s, 10 ms, 0.7	Inhibitory gating variables
γ	0.641/1000	Kinetic parameter
σ	0.01 nA	Noise amplitude
I_0	0.382 nA	Overall effective external input
C_{ij}	Obtained from diffusion tractography	Structural connectivity (SC) matrix
G	Obtained from parameters optimization	Global coupling scaling factor
J_i	Obtained from parameters optimization	Feedback inhibitory synaptic coupling
J_{NMDA}	Obtained from parameters optimization	Excitatory synaptic coupling
w_+	Obtained from parameters optimization	Local excitatory recurrence
